# Celastrol: An Update on Its Hepatoprotective Properties and the Linked Molecular Mechanisms

**DOI:** 10.3389/fphar.2022.857956

**Published:** 2022-04-04

**Authors:** Mengzhen Li, Faren Xie, Lu Wang, Guoxue Zhu, Lian-Wen Qi, Shujun Jiang

**Affiliations:** ^1^ Clinical Metabolomics Center, China Pharmaceutical University, Nanjing, China; ^2^ Nanjing Hospital of Chinese Medicine Affiliated to Nanjing University of Chinese Medicine, Nanjing, China

**Keywords:** celastrol, liver diseases, liver injury, liver cancer, MAFLD

## Abstract

The liver plays an important role in glucose and lipid homeostasis, drug metabolism, and bile synthesis. Metabolic disorder and inflammation synergistically contribute to the pathogenesis of numerous liver diseases, such as metabolic-associated fatty liver disease (MAFLD), liver injury, and liver cancer. Celastrol, a triterpene derived from *Tripterygium wilfordii* Hook.f., has been extensively studied in metabolic and inflammatory diseases during the last several decades. Here we comprehensively review the pharmacological activities and the underlying mechanisms of celastrol in the prevention and treatment of liver diseases including MAFLD, liver injury, and liver cancer. In addition, we also discuss the importance of novel methodologies and perspectives for the drug development of celastrol. Although celastrol has been claimed as a promising agent against several metabolic diseases, both preclinical and clinical studies are highly required to accelerate the clinical transformation of celastrol in treating different liver illness. It is foreseeable that celastrol-derived therapeutics is evolving in the field of liver ailments.

## Introduction

The liver is a metabolic organ that is responsible for glucose and lipid homeostasis, drug metabolism, and bile synthesis (Diehl-Jones and Askin, 2002; Almazroo et al., 2017; Tappy, 2021). Liver diseases contain non-alcoholic fatty liver disease (NAFLD) and its related diseases, drugs or infections induced liver injury, liver cancer, and among others. With the aging of the population and the prevalence of obesity, NAFLD, known as metabolic-associated fatty liver disease (MAFLD), is becoming a threat to human health in the world, (Eslam et al., 2020). MAFLD begins with the accumulation of hepatic lipid, metabolic syndrome (central adiposity, hyperglycemia, dyslipidemia, and hypertension) (Mundi et al., 2020). Environment factors like dietary habits and the genetic factors such as *PNPLA3* (encoding patatin-like phospholipase domain-containing protein3), *TM6SF2* (encoding transmembrane 6 superfamily member 2), and epigenetic factors are the risk factors of MAFLD (Younossi et al., 2018). The current global prevalence of MAFLD is approximately 25%, which may develop into non-alcoholic steatohepatitis, eventually leading to liver fibrosis, cirrhosis, and even liver cancer if not treated in a timely fashion (Ye et al., 2020). Except for the liver damage, MAFLD and non-alcoholic steatohepatitis can also aggravate or induce insulin resistance, which are closely related to the high incidence of type 2 diabetes and cardiovascular disease (Liang et al., 2022). Liver injury as a loss of liver function is mainly caused by drug, infections, and intrahepatic cholestasis (Thawley, 2017). The drug-induced liver injury (DILI) is one of the most common and serious adverse drug reactions, which can lead to acute liver failure and even death in severe cases (Licata, 2016). With 831,000 death cases and 905,000 new cases in 2020, liver cancer with high mortality and incidence remains a global menace (Sung et al., 2021). Although many patients are diagnosed and treated at an early stage of the disease, the recurrence rate is still high. For middle and late-stage hepatic carcinoma, the prognosis is even less optimistic. Therefore, new therapy or drugs with better efficacy and lower toxicity are of great importance.

Traditional Chinese Medicine (TCM) was used to treat various diseases such as metabolic diseases, liver diseases, inflammation, and cancers for many years. In light of their numerous pharmacological activities, low toxicity, and low side effects, nature compounds from TCM are widely used to protect against liver diseases, such as berberine, curcumin, ginsenoside, celastrol, to name a few (Xu et al., 2018a). In Europe and America, up to 65% of patients choose herbal medicines to treat liver diseases (Zhang et al., 2013; Madrigal-Santillán et al., 2014; Bagherniya et al., 2018). Celastrol, a pharmacologically active compound from *Tripterygium wilfordii* Hook.f., possesses therapeutic properties for multiple diseases, such as rheumatoid arthritis, inflammatory bowel diseases, hepatitis, as well as anti-cancer, anti-obesity, and metabolic modulating (Feng et al., 2013; Lin et al., 2015; Liu et al., 2015; Tseng et al., 2017; Saito et al., 2019; Song et al., 2019). In recent years, celastrol has attracted the attention of researchers and the pharmaceutical industry due to its benefits on human health. Here, we summarized the studies exploring the pharmacological properties of celastrol in liver protection during 15 years from 2007 to 2021, and made a general statement about its mechanisms in liver-related diseases.

## Study Design

**FIGURE 1:**
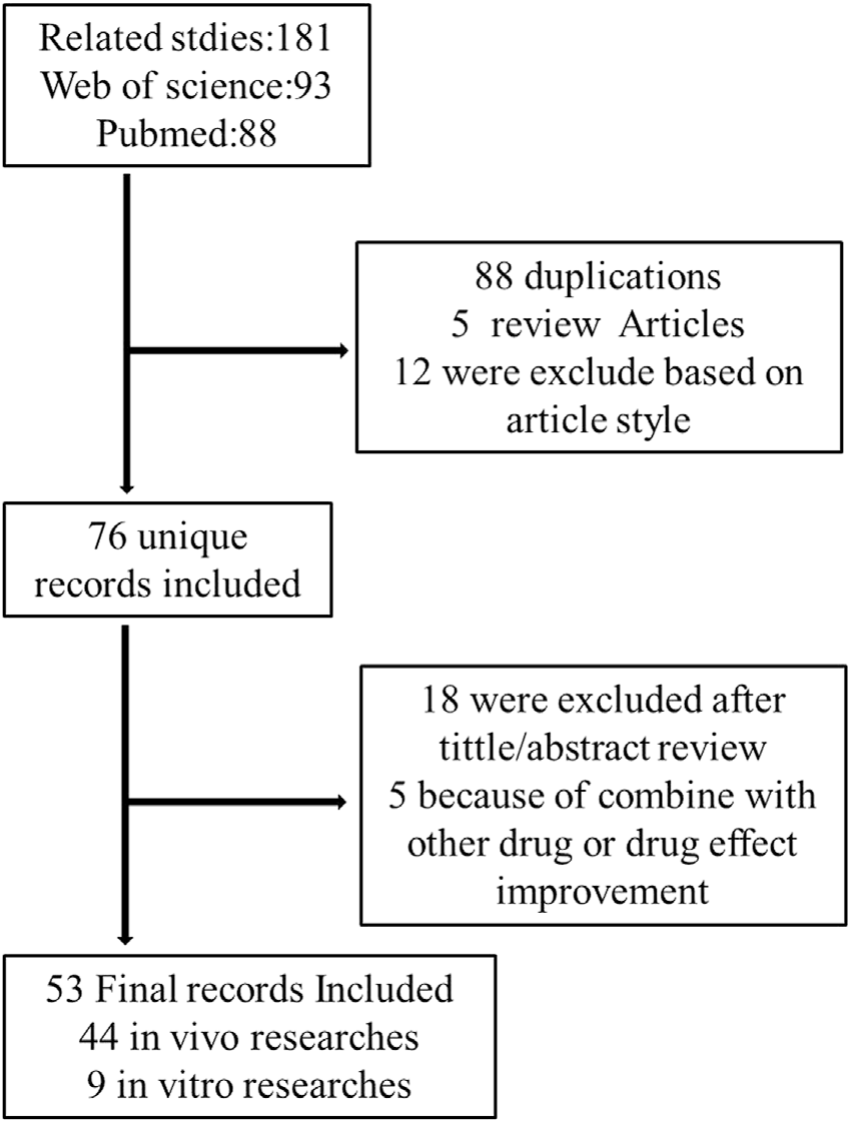
Flow chart of the study design.

The PubMed and Web of Science databases were used to search related articles from 2007 to 2021. Cellular, animal, or human studies were searched with the keywords (celastrol) and (liver). Papers were extracted using the following inclusion criteria: (1) these studies should be research articles to estimate the hepatoprotective effects of celastrol; (2) only articles written in English were included. All titles and abstracts of publications retrieved were reviewed to select potentially eligible studies. Full texts of literatures potentially eligible were reviewed by two independent investigators. From a total of 181 results, 76 unique studies were identified, 88 duplication, 5 reviews, and 12 other article styles were excluded. After carefully reviewing the title and/or abstract, 18 were excluded 5 publications are irrelevant. A total of 53 articles were included in this review. A flowchart of this is illustrated in Figure 1.

## Celastrol and Liver Diseases

### Celastrol and MAFLD

MAFLD, characterized by the hepatic fat accumulation, is usually correlated with the metabolic disorder related obesity (Samuel and Shulman, 2018; Jarvis et al., 2020), hepatic inflammation (Kazankov et al., 2019), oxidative stress (Ore and Akinloye, 2019; Świderska et al., 2019), and intestinal microbiome dysbiosis (Caussy and Loomba, 2018). Lipotoxicity induced by abnormal hepatic fat deposition activated Kuepfer cell (KC) to promote inflammation in MAFLD patients (Lanthier, 2015). Oxidative stress and endoplasmic reticulum (ER) stress resulted in mitochondrial reactive oxygen species (ROS) accumulation in hepatocytes, which in turn impaired glycolipid metabolism, accelerated inflammatory response, and introduced cell death in MAFLD (Ore and Akinloye, 2019; Świderska et al., 2019; Chen et al., 2020a). The reasonable management of body weight and dietary habits should be given a high priority in MAFLD patients. Although the antidiabetic and lipid-lowering drugs may help to reduce hepatic lipid accumulation (Mundi et al., 2020), there are no specific drugs for MAFLD approved in Europe and the United States. Recent research mainly focused on the metabolic targets, immune targets, fibrosis, cell stress, and apoptosis (Friedman et al., 2018). Celastrol as a promising compound exhibits good anti-obesity and anti-inflammatory effect on treating MAFLD.

#### Anti-Obesity Effects

The liver is vital for lipid and glucose metabolism and homeostasis, protein, and amino acid metabolism, where bile acids produce and flux to the digestive system. Numerous evidences supported that obesity is strongly associated with MAFLD development (Fabbrini et al., 2010). The liver protective effect and molecular mechanism of celastrol on high fat diet (HFD) induced MAFLD animal models have been well-investigated (Wang et al., 2014; Ma et al., 2015; Zhang et al., 2017a; Hu et al., 2017; Luo et al., 2017; Kyriakou et al., 2018; Pfuhlmann et al., 2018; Zhang et al., 2018; Feng et al., 2019a; Zhao et al., 2019a; Feng et al., 2019b; Chellappa et al., 2019; Fang et al., 2019; Abu Bakar et al., 2020; Hu et al., 2020; Zhu C. et al., 2021; Zhu Y. et al., 2021; Hua et al., 2021; Ouyang et al., 2021; Yang et al., 2021).

As a typical feature of obesity, leptin resistance was involved in the hepatic steatosis during the development of MAFLD (Myers et al., 2010; Boutari and Mantzoros, 2020). Leptin, a hormone secreted by adipose tissue, directly interacted with the leptin receptor in the brain and mediated food intake, energy metabolism, body weight, and other physiological processes (Xu et al., 2018b; Caron et al., 2018; Pan and Myers, 2018). Brain leptin promoted hepatic lipid flux and reduced lipogenesis in the liver (Hackl et al., 2019). Celastrol as a leptin sensitizer exhibited significant weight loss and food consumption reduction in HFD-obesity animals (Wang et al., 2014; Liu et al., 2015; Ma et al., 2015; Zhang et al., 2017a; Hu et al., 2017; Luo et al., 2017; Kyriakou et al., 2018; Pfuhlmann et al., 2018; Zhang et al., 2018; Feng et al., 2019a; Zhao et al., 2019a; Feng et al., 2019b; Chellappa et al., 2019; Fang et al., 2019; Saito et al., 2019; Abu Bakar et al., 2020; Hu et al., 2020; Zhu C. et al., 2021; Zhu Y. et al., 2021; Hua et al., 2021; Ouyang et al., 2021; Yang et al., 2021) (Table 1).

**TABLE 1:** The summary of preclinical studies of celastrol in HFD induced MAFLD models.

Experimental model used	Model inducer	Dosage and drug-delivery way	Drug treatment period	Phenotype and mechanism	Reference
Wild type animal models					
Male Sprague–Dawley rats	HFD	1 mg/kg/day, 3 mg/kg/day, and 9 mg/kg/day by oral administration	6 weeks	Promoted weight loss and lipid metabolism, attenuated oxidative injury through improving ABCA1 and antioxidant enzymes activities, reducing NADPH oxidase activity	Wang et al. (2014)
	HFD for 11 weeks	500 μg/kg/day by oral administration	3 weeks	Decreased body weight and lipid accumulation in liver, promoted energy expenditure by increasing ratio of *Bacteroidetes* to *Firmicutes* rather than food intake, leptin signaling pathway, gut microbiota homeostasis	Hu et al. (2020)
	HFD for 17 weeks	1 mg/kg/day, 3 mg/kg/day mixed with drinking water	8 weeks	Reduced body weight, alleviated inflammatory response in adipose tissue and enhanced mitochondrial functions in skeletal muscle by upregulation of AMPK/SIRT1 signaling pathways	Abu Bakar et al. (2020)
Male C57BL/6J mice	HFD for16-20 weeks	100 μg/kg/day by i.p injection, or 10 mg/kg/day by oral administration	3 weeks	Improved weight loss and glucose homeostasis by reducing food consumption and ER stress in hypothalamus	Liu et al. (2015)
	NCD or HFD for 9 weeks	100 μg/kg/day or 500 μg/kg/day by i.p injection	24 days	Reduced body weight and fat mass by decreased food intake, improved metabolism by increased homeostatic regulation of energy balance related gene expressions in the hypothalamus	Saito et al. (2019)
	HFD for 8 weeks	1 mg/kg/day, 3 mg/kg/day mixed with food	3 weeks	Enhanced energy expenditure, and mitochondrial function in fat and muscle by activated HSF1-PGC1α axis	Ma et al. (2015)
	HFD for 14 weeks	200 μg/kg/every 2 days by i.p injection	4 weeks	Inhibited lipid synthesis by downregulation of Srebp-1c expression, reduced oxidative stress and inflammation by enhanced the phosphorylation of hepatic AMPKα and Sirt1	Zhang et al. (2017a)
	HFD for 12 weeks	100 μg/kg/day by i.p injection	2 weeks	Suppressed hepatic inflammation and immune cell accumulation by reducing expression and production of IL-1β and IL-6	Hu et al. (2017)
	HFD for 16 weeks	100 μg/kg/day by i.p injection	8 weeks	Attenuated inflammation and insulin resistance by inhibition of TLR4/NF-κB	Zhang et al. (2018)
	HFD for 32 weeks	100 μg/kg/day by i.p injection	6 days	Promoted weight loss through hypoplasia and activation of leptin-STAT3 signaling in elder mice	Pfuhlmann et al. (2018)
	HFD for 8–12 weeks	100 μg/kg/day by i.p injection	10 days	Lowered body weight by inhibition of PTP1B and TCPTP in hypothalamus	Kyriakou et al. (2018)
	HFD for16-20 weeks	100 μg/kg/day by i.p injection	4 days	Celastrol’s anti-obesity effects was not dependent on LCN2	Feng et al. (2019a)
	HFD for 16 weeks	0.1 mg/kg/day by i.p injection	21 days	Suppressed gluconeogesis by activating CREB/PGC-1α pathway	Fang et al. (2019)
	HFD for 12 weeks	150 μg/kg, 300 μg/kg by i.p injection	3 weeks	Reduced weight gain without affecting food intake, ameliorated metabolic disorder and hepatic inflammation by inhibition of TLR3/NLRP3 inflammasome	Yang et al. (2021)
	HFD for 6 weeks	3 mg/kg/day was mixed with food	24 days	Prevented intestinal lipid absorption by downregulation of CD36, FATP2, FATP4	Hua et al. (2021)
	HFD for 12 weeks	50 μg/kg/day, 100 μg/kg/day, 200 μg/kg/day by i.p injection	12 weeks	Attenuated inflammation through the suppression of MMP-2 and MMP-9	Ouyang et al. (2021)
	HFD for 16 weeks	0.75 mg/kg/day,1.5 mg/kg/day,3 mg/kg/day by oral administration	25 days	Reduced body weight gain, insulin resistance, hepatic steatosis, and inflammation by inhibition of CAP1‒resistin interaction, PKA‒NF-kB pathway	Zhu Y. et al. (2021)
Male C57BL/6 N mice	HFD for 12 weeks	5 mg/kg/day and 7.5 mg/kg/day mixed with food	3 weeks	Prevented M1 macrophage polarization, inflammation, and insulin resistance via regulating Nrf2/HO‐1, MAPK signal, and NF-κB pathway	Luo et al. (2017)
	HFD for12 weeks	5 mg/kg/day or 7.5 mg/kg/day by oral administration	3 weeks	Reduced body weight and fat mass inhibited inflammatory response by downregulation of expression of macrophage M1 biomarkers (e.g., IL-6, IL-1β, TNF-α, iNOS) and enhanced expression of macrophage M2 biomarkers (e.g., Arg-1, IL-10)	Zhao et al. (2019a)
Genetic deficency animal models					
Lep^db^ mice	NCD	100 μg/kg/day by i.p. injection, or 10 mg/kg/day by oral administration	3 weeks	No significant change of body weight	Liu et al. (2015)
		100 μg/kg/day by subcutaneous injection	6 days		Pfuhlmann et al. (2018)
		100 μg/kg by i.p. injection	3 weeks		Feng et al. (2019b)
		100 μg/kg by i.p. injection	4 days		Feng et al. (2019a)
		0.1 mg/kg/day by i.p. injection for 10 days, then 0.5 mg/kg by i.p.injection for 15 days	25 days	Body weight slightly reduced	(Saito et al., 2019)]
Lep^−/+^ rats and Lep^−/−^ rats	HFD for 17 weeks	0.5 mg/kg/day or 1 mg/kg/day by oral administration	3 weeks	1,000 μg/kg celastrol decreased the BW of Lep^−/+^ rats not Lep^−/−^ rats	Hu et al. (2020)
Lep^ob^ mice	NCD for 6 or 14 weeks	100 μg/kg/day by subcutaneous injection	6 days	Promoted weight loss in young Lep^ob^ mice not old Lep^ob^ mice	Liu et al. (2015)
HSF1 ^−/−^ Mice	HFD for 4 weeks	3 mg/kg/day mixed with powdered chow	4 weeks	Had no effects on body weight and energy consumption	Ma et al. (2015)
Liver specific Sirt1 KO mice	HFD for 14 weeks	200 μg/kg/every 2 days by i.p.injection	4 weeks	Reduced food intake and increased the hepatic lipid accumulation by inhibited phosphorylation of AMPKα and hepatic LKB1 expression	Zhang et al. (2017a)
Nur77 −/−mice	HFD for 17 weeks	0.1 mg/kg/day by i.p injection	2 weeks	Mild reduced the body weight and anti-inflammation effects attenuated	Hu et al. (2017)
Global PTP1B KO mice	NCD or HFD for 10 weeks	0.1 mg/kg/day by i.p injection	7 days	Induced weight loss both in NCD and HFD PTP1B mice, reduction of fat and lean mass is owing to weight loss of HFD PTP1B mice not for NCD mice	Pfuhlmann et al. (2018)
UCP1 KO mice	HFD for 20 weeks	100 μg/kg/day by subcutaneous injection	6 days	Decreased body weight and food intake by fat mass loss	Pfuhlmann et al. (2018)
IL1R1^−/−^ mice	HFD for 20 weeks	100 μg/kg/day by i.p. injection	3 weeks	No change of body weight, fat mass, and food intake	Zhao et al. (2019a)
Lcn2^−/−^ mice	NCD or HFD for 16-20 weeks	100 μg/kg/day by i.p. injection	3 weeks	Reduced body weight and fat mass without affected food intake in NCD Lcn2^−/−^ mice, inhibited hepatosteatosis, and metabolic disorder induce by HFD in Lcn2^−/−^ mice	Feng et al. (2019a)
ApoE^−/-^ mice	NCD or HFD for 12 weeks	100 μg/kg/day by oral administration	12 weeks	Alleviated inflammatory reaction in apoE^−/-^ mice fed with HFD	Zhu Y. et al. (2021)
Melanocortin 4 receptor (MC4R)-null mice	NCD	0.1 mg/kg/day by i.p injection for 10 days, then 0.5 mg/kg/day by i.p injection for 15 days	25 days	Reduced body weight, food intake, fat and lean mass, enhanced energy expenditure by upregulation of adrenergic receptor and PRDM16 without affecting UCP-1 and PGC-1α	Saito et al. (2019)
HnRNPA1 deficency/overexpression mice	NCD	2 mg/kg/day by gavage administration	12 days	Inhibited energy expenditure and abrogated weight loss effects in HnRNPA1 overexpression mice	Zhu C. et al. (2021)
Other model					
Young (4–6 month) and Old (18–22 month) male mice	NCD	100–200 μg/kg/day by i.p. injection	4–6 days	Promoted weight and lean mass loss by lowering food intake in aged mice, but not in young controls	Chellappa et al. (2019)

HFE, high fat emulsion; NCD, normal chow diet; HFD, high fat diet; ABCA1, ATP-binding cassette transporter A1; NADPH, nicotinamide adenine dinucleotide phosphate; AMPK, Adenosine 5‘-monophosphate (AMP)-activated protein kinase; SIRT1, sirtuin1; ER, endoplasmic reticulum; HSF1, heat shock factor 1; PGC-1α, Peroxisome proliferator-activated receptor γ coactivator 1α; Srebp-1c, sterol regulatory element binding protein-1c; IL-1β, interleukin-1β; IL-6, interleukin-6; TLR4, Toll-like receptor 4; NF-κB, nuclear factor kappa-B; STAT3, signal transducer and activator of transcription 3; LCN2, lipocalin-2; PTP1B, protein tyrosine phosphatase (PTP) 1B; TCPTP,T-cell PTP; TLR3, Toll-like receptor 3; NLRP3, NOD-like receptor protein 3; CD36, cluster of differentiation 36; FATP2, very-long-chain acyl-CoA, synthetase; FATP4, fatty acid transport protein 4; MMP-2, Matrix metalloproteinase-2; MMP-9, Matrix metalloproteinase-9; CAP1, adenylate cyclase-associated protein 1; PKA, Protein kinase A; NF-kB, nuclear factor kappa B; Nrf2, nuclear respiratory factor 1; HO-1, Heme oxygenase 1; MAPK, mitogen-activated protein kinase; TNF-α, tumor necrosis factor α, iNOS, inducible nitric oxide synthase; Arg-1, arginase-1; IL-10, interleukin-10; Lep, leptin; BW, body weight; LKB1, liver kinase B1; PTP1B, Protein tyrosine phosphatase 1; ApoE, apolipoproteinE; PRDM16, PR, domain-containing 16; UCP-1, Uncoupling protein 1; HnRNPA1,heterogeneous nuclear ribonucleoprotein A1.

Celastrol (100 μg/kg by intraperitoneally injection [i.p], or 10 mg/kg by oral administration [OA]) increased the leptin sensitivity to suppress food intake and reduce body weight in HFD induced C57BL/6J mice and was firstly reported in 2015 by Ozcan’s group (Liu et al., 2015), while the benefits of celastrol on obesity were abrogated in lean mice, leptin receptor-deficient *(db/db),* or leptin-deficient *(ob/ob)* mice/rats (Liu et al., 2015; Pfuhlmann et al., 2018; Feng et al., 2019a; Feng et al., 2019b; Saito et al., 2019; Hu et al., 2020). Compared with leptin or celastrol alone, the combined use of leptin and celastrol could significantly inhibit appetite and weight gain of different mice, even for *ob/ob* mice (Liu et al., 2015). Numerous studies verified the effect of leptin sensitizer celastrol on appetite as showing in Table 1.

The underlying molecular mechanism of celastrol anti-obesity and increase the leptin sensitivity can be attributed to Sirtuin 1(Sirt1) (Zhang et al., 2017a; Abu Bakar et al., 2020), interleukin-1 receptor 1(IL1R1) (Feng et al., 2019b), protein tyrosine phosphatase (PTP) 1B (PTP1B), and T-cell PTP (TCPTP) (Kyriakou et al., 2018). SIRT1 as a deacetylase activated by energy deprivation was involved in glucose, lipid, and bile acid metabolism regulation (Purushotham et al., 2009; García-Rodríguez et al., 2014). It has been reported that celastrol exacerbated liver metabolic disorder by suppressing the phosphorylation of AMP-activated protein kinase α (AMPKα) in liver specific Sirt1 knock out mice fed with HFD (Zhang et al., 2017a). In line with that, celastrol (3 mg/kg/day for 8 weeks) significantly increased insulin sensitivity and weight loss through enhancing AMPK/SIRT1 signaling in HFD-induced rats (Abu Bakar et al., 2020). IL1R1, a cytokine receptor, was associated with cell death and inflammation. IL1R1 deficient mice showed the phenotype of mature-onset obesity and leptin resistance (García et al., 2006). Feng and his colleagues demonstrated that the protective property of celastrol on obesity reversed by IL1R1 deficiency, which indicated that IL1R1 is essential for celastrol to reduce food consumption and body weight, alleviate glucose intolerance and insulin tolerance, as well as hepatic steatosis in HFD mice (Feng et al., 2019). However, these studies were only performed in genetic deficient animals, and whether the hepatoprotective effects of celastrol depended on its direct interaction with Sirt1 or IL1R1 need further studies. The surface plasmon resonance (SPR) assay, circular dichroism (CD) spectroscopy, and other methods might be effective methods to verify the above hypothesis. PTP1B and TCPTP negatively regulate leptin signaling in the hypothalamus (Dodd et al., 2019). Eleni et al. demonstrated that celastrol-induced weight loss was attributed to the supression of PTP1B and TCPTP, and inhibition of PTP1B and TCPTP is mediated by reversible noncompetitive binding to an allosteric pocket close to the active site (Kyriakou et al., 2018). In addition, different studies demonstrated the anti-obesity effect of celastrol was independent on melanocortin 4 receptor (MC4R), and lipocalin 2 (Lcn2) (Feng et al., 2019a; Saito et al., 2019), because the deletion of these genes did not weaken the weight-loss and liver-protecting effect of celastrol in mice.

Obesity related MAFLD was triggered by energy imbalance, with features of excessive lipid deposition. Peroxisome proliferator-activated receptor γ coactivator 1α (PGC-1α), a nuclear transcriptional coactivator factor, increased energy expenditure by regulating mitochondrial function and biogenesis (Vega et al., 2000; Rodgers et al., 2005; Minsky and Roeder, 2015). Liver-specific deficient PGC-1α mice exhibit hepatic steatosis (Rodgers et al., 2005). Accumulating evidences uncovered that celastrol promotes the white adipose tissue (iWAT) browning and brown adipose tissue (BAT) activation by upregulating PGC-1α and uncoupling protein 1(UCP1, a downstream effector of PGC-1α, is responsible for electron transport of mitochondrial oxidative phosphorylation) expression (Table 2). A total of 3 mg/kg/day celastrol (OA) significantly increased the HSF1 (Heat Shock Transcription Factor 1)/PGC-1α axis activity and protected against obesity in HFD mice by increasing energy expenditure, including iWAT browning, BAT activation, and mitochondrial gene transcription, while such benefit was abrogated by HSF1 depletion (Ma et al., 2015). In agreement with this result, several reports also clarified that celastrol increased the energy expenditure by activating iWAT browning through upregulation of PGC-1α and UCP1 (Fang et al., 2019; Yang et al., 2021). However, these studies were challenged by Katrin’s group, and they showed that celastrol intervention only upregulated the UCP1, while the expression of PGC-1α in BAT of HFD mice was not altered, and UCP1 deletion in HFD mice did not abolish food consumption and body weight reduction effects of celastrol (Pfuhlmann et al., 2018). This indicated that weight loss effect of celastrol was not dependent on UCP1-mediated thermogenesis under nutrient stress. In accordance with Katrin’s study, other researchers did not observe that the upregulation of UCP1 and PGC-1α expression in iWAT and BAT of HFD induced mice and rats with celastrol administration (Saito et al., 2019; Hu et al., 2020; Hua et al., 2021). The disparity of above observations of celastrol’s hepatoprotective effects might be caused by different celastrol-delivery way (OA vs. i.p), dosage, and intervention time period (Table 2). All above results indicated that iWAT browning happened at a specific time after celastrol treatment, and celastrol improved energy expenditure through other pathways not only depending on browning of iWAT.

**TABLE 2:** The regulation of celastrol on iWAT browning and BAT thermogenesis gene.

Animal model	Model inducer	Dosage	Intervention time	Gene		Reference
				UCP-1	PGC-1α	
Male Sprague–Dawley rats	HFD for 11 weeks	0.5 mg/kg/day by oral administration	3 weeks	no change	no change	Hu et al. (2020)
C57BL/6J	NCD or HFD for 9 weeks	0.1 mg/kg/day or 0.5 mg/kg/day by i.p injection	24 days	no change	no change	Saito et al. (2019)
	HFD for 6 weeks	3 mg/kg/day celastrol was mixed with food	24 days	no change	no change	Hua et al. (2021)
	HFD for 8 weeks	1 mg/kg/day, 3 mg/kg/day mixed with food	3 weeks	upregulated	upregulated	Ma et al. (2015)
	HFD for 12 weeks	0.1 mg/kg/day,0.3 mg/kg/day by i.p injection	3 weeks	upregulated	upregulated	Yang et al. (2021)
	HFD for 16 weeks	0.1 mg/kg/day by i.p injection	21 days	upregulated	upregulated	Fang et al. (2019)
	HFD for 32 weeks	0.1 mg/kg/day by i.p injection	6 days	upregulated	no change	Pfuhlmann et al. (2018)
MC4R-null mice	NCD	0.1 mg/kg/d by i.p injection for 10 days, then 0.5 mg/kg/d by i.p injection for 15 days	25 days	no change	no change	Saito et al. (2019)
HSF1 ^−/−^ mice	NCD for 8 weeks	3 mg/kg/day mixed with food	4 weeks	no change	no change	Ma et al. (2015)

iWAT, inguinal white adipose tissue; BAT, brown adipose tissue; SD, Sprague–Dawley; UCP-1, Uncoupling protein 1; PGC-1α, Peroxisome proliferator-activated receptor γ coactivator 1α; MC4R, melanocortin 4 receptor; HSF1, heat shock factor 1; NCD, normal chow diet; HFD, high fat diet.

Numerous studies proved that gut microbiota dysbiosis can also be attributed to fatty liver disease development (Le Roy et al., 2013; Canfora et al., 2019; Frazier and Chang, 2020). Oral administration of 500 μg/kg/day celastrol markedly enhanced energy expenditure and enhanced liver lipid metabolism in HFD rats by improving the gut microbiota homeostasis and activating the hypothalamic leptin signaling pathway rather than affecting food intake (Hu et al., 2020). *Firmicutes* promoted lipid absorption not fatty acid oxidation (Murphy et al., 2013). Increased ratio of *Bacteroidetes* to *Firmicutes* played a critical role in improvement of lipid consumption in celastrol-treated HFD rats (Murphy et al., 2013). Consistent with this, oral administration of celastrol (3 mg/kg/d) inhibited intestinal lipid absorption to ameliorate metabolic disturbance by reconstructing the gut microbiota profile in HFD mice (Hua et al., 2021). Although it has been reported that celastrol improved lipid metabolism by modulating gut microbiota, its specific mechanism of gut microbiota involved in MAFLD still needs to be explored. In addition, recent study demonstrated that age-associated obesity mice injected with celastrol (100–200 μg/kg) exhibited reduction body weight and fasting glucose level owing to fat loss and circadian rhythms restored without impact on lean mass (Chellappa et al., 2019), and whether gut microbiota act as an actuator of host circadian rhythms involved in the procedure remains to be studied (Frazier and Chang, 2020; Saran et al., 2020).

In summary, celastrol reduced body weight and hepatic fat accumulation mainly by increasing leptin sensitivity, enhancing energy metabolism, and modulating gut microbiota. Above all, Sirt1, IL1R1, PTP1B, and TCPTP might be the potential targets of celastrol, but whether celastrol directly interacted with these targets should be deeply explored. Moreover, the effect of celastrol on PGC-1α and UCP1 is controversial, and further studies should be performed to verify this effect. Gut microbiota have been extensively studied in recent years, but the molecular mechanism of celastrol on anti-obesity through gut microbiota is still unclear.

#### Anti-Inflammatory Response Activity

Macrophage infiltration and inflammation caused by dyslipidemia is associated with progression of MAFLD (Kazankov et al., 2019; Katsiki et al., 2016; Luo et al., 2018). Different animal experiments have elucidated that the inflammation in fatty liver mice treated with celastrol are remarkably attenuated (Table 1). Macrophage infiltration and inflammatory factors (IL-1β, IL-18, MCP-1α, and TNF-α) were reduced in liver of HFD induced C57BL/6J or C57BL/6N mice by celastrol treated for 3 weeks (Hu et al., 2017; Luo et al., 2017; Zhang et al., 2018; Ouyang et al., 2021; Zhu Y. et al., 2021). In addition, adipose tissue inflammation and mitochondrial dysfunction in skeletal muscle ameliorated in HFD-induced SD rats treated with 3 mg/kg/day celastrol for 8 weeks (Abu Bakar et al., 2020). The mechanism exhibited that celastrol can activate AMPK/SIRT1 signaling pathways for mitochondrial function improvement and attenuate inflammatory responses via decreasing nuclear factor kappa-B (NF-κB) activity (Abu Bakar et al., 2020). Celastrol-mediated hepatoprotective effects in C57BL/6J mice fed with HFD abolished in liver specific Sirt1-deficient mice fed HFD (Zhang et al., 2017a). Celastrol directly interacted with adenylyl cyclase associated protein 1 (CAP1) in macrophages contributed to improvement of metabolism and attenuation of inflammatory response through NF-κB signaling pathway in HFD-induced mice (Zhu Y. et al., 2021). Apart from that, LPS induced acute liver inflammation and HFD induced chronic inflammation are both inhibited by celatrol through binding Nur77 and promoting Nur77 interact with TRAF2 inducing autophagy (Hu et al., 2017). Deletion of Nur77 or Sirt1 impaired the anti-inflammation ability of celastrol, but LCN2 or ApoE deficiency did not influence the anti-hepatosteatosis of celastrol (Hu et al., 2017; Zhang et al., 2017a; Feng et al., 2019b; Ouyang et al., 2021). In general, the protective effects of celastrol against inflammation might be attributed to suppression of NF-κB involved signaling pathway.

**FIGURE 2:**
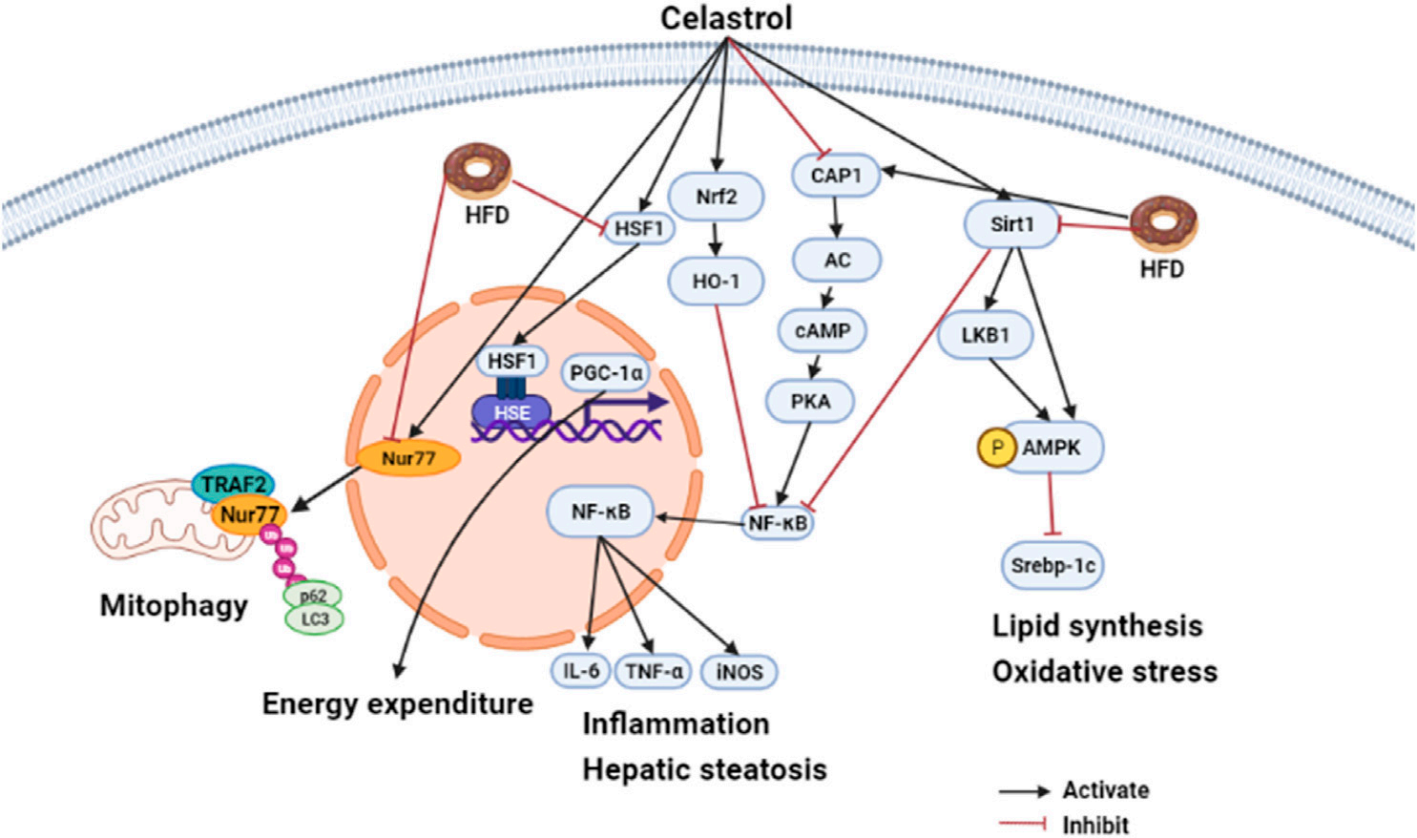
Hepatoprotective effects of celastrol in HFD induced animal model (edited by Biorender software). Diagram of the mechanism of celastrol in regulating hepatic energy expenditure, inflammation, and lipid metabolism. Under HFD stress, celastrol attenuated inflammation by inducing Nur77 interaction with TRAF2 to promote mitochondrial autophagy. Macrophages mediated inflammation was also ameliorated by celastrol targeted CAP1 via inhibition of cAMP-PKA-NF-κB signaling pathway, and macrophage M1 polarization was suppressed by celastrol via regulating Nrf2-HO-1-NF-κB pathways. In addition, celastrol can also regulate metabolism disorder. On the one hand, it can promote energy metabolism by activating HSF1-PGC1*α*; on the other hand, it can target SIRT1 to promote AMPK *α* phosphorylation and inhibit Srebp-1c-mediated lipid synthesis against oxidative stress and inflammation TRAF2, TNFR-associated factor2; CAP1, adenylyl cyclase-associated; protein 1 cAMP, cyclic adenylate monophosphate; PKA, protein kinase A; NF-κB, nuclear factor kappa-B; Srebp-1c, sterol regulatory element binding protein 1c; AMPK*α*, AMP-activated protein kinase *α*.

In summary, different animal experiments confirmed that celastrol had metabolic improvement effect on HFD induced SD rats/mice or aged-obesity mice, but has no effect on gene deficiency mice such as HSF1 deletion mice, IL1R1 null mice, Sirt1 deficient mice, etc. (Ma et al., 2015; Zhang et al., 2017a; Feng et al., 2019b). This indicated that HSF1, IL1R1, and Sirt1 might act as the target of celastrol. In addition, oral administration of celastrol hardly affects food intake but has the same benefit on metabolic compared to intraperitoneal injection. Therefore, it is better to use oral administration to explore the pharmacological mechanism of celastrol. The effects of celastrol on MAFLD are summarized in Figure 2.

### Celastrol and Liver Injury

Liver injury characterized by a loss of hepatocyte function in patients resulted from mitochondrial oxidative stress, endoplasmic reticulum stress, inflammation, and autophagy in hepatocyte (Vento and Cainelli, 2020). Drugs, infections, and intrahepatic cholestasis can all lead to liver injury (Fontana et al., 2014). Celastrol, an anti-inflammatory drug, ameliorated the damage in hepatocyte or liver injury (Abdelaziz et al., 2017; El-Tanbouly et al., 2017; Jannuzzi et al., 2018; Wu et al., 2018; Zhao et al., 2019b; Guo et al., 2019; Wang et al., 2020; Zhao et al., 2020; Yan et al., 2021) (Table 3).

**TABLE 3:** Pharmacological activities of celastrol in the treatment of liver injury.

Model style	Cell/animal	Inducer	Dosage of celastrol	Treatment time	Phenotype/mechanism	Reference
Chemicals induced liver injury	HepG2 cells	APAP	50, 100, and 200 nM	24 h	Ameliorated oxidative stress and cytotoxicity caused by APAP	Jannuzzi et al. (2018)
Chemicals induced liver injury	Male BALB/c mice	APAP	2 mg/kg by i.p injection	2 h prior APAP-induction	Prevented oxidative stress and inflammation by attenuating inflammatory cells accumulation and reducing inflammation factors	Abdelaziz et al. (2017)
Chemicals induced liver injury	Male WT mice and Ppara^−/−^ mice on the 129/Sv genetic background	CCl4	10 mg/kg by oral treatment	5 days	Inhibited inflammatory cytokine and oxidative stress by suppressing PPARα signaling pathway, the effects of celastrol attenuated DCA-EGR1-inflammatory factor signaling in CCl4-induced PPARα deleted mice	Zhao et al. (2020)
Chemicals induced liver injury	Male Sprague	CCl4	0.25 mg/kg/day,0.5 mg/kg/day,1 mg/kg/day by i.p injection	4 weeks	Suppressed inflammation in liver fibrosis by activating AMPK-SIRT3 signaling	Wang et al. (2020)
	Dawley rats					
Cholestatic liver injury	Male C57BL/6J mice	ANIT	10 mg/kg/day by oral administration	5 days	Alleviated cholestatic liver injury by activation SIRT1-FXR signaling pathway	Zhao et al. (2019b)
	Male C57BL/6J mice and Fxr-null mice	TAA			FXR disruption attenuated protection effects of celastrol on cholestatic liver injury	
Cholestatic liver injury	Female Sprague-Dawley rats	EE	5 mg/kg/day by an oral administration	5 days	Alleviated phenotype of ICP by inhibited MMP-2 and MMP-9	Guo et al. (2019)
Sepsis induced liver injury	Male Sprague Dawley rats	CLP	1 mg/kg by i.p injection	60 min before CLP	Attenuated inflammation by suppressing NF- κB, reduced TLR-4 and 5-LOX expression, downregulated the expression of IL-6	El-Tanbouly et al. (2017)
Sepsis induced liver injury	Male C57BL/6 mice	LPS	1.5 mg/kg/day by i.p injection	24 h before LPS induction, after LPS intoxiant for another 24 h	Aggravated liver injury through activating inflammation and deteriorating oxidative stress	Wu et al. (2018)
Sepsis induced liver injury	Male C57BL/6J mice and NLRP3-/- mice	P. acnes/LPS	0.5 or 0.25 mg/kg by i.p injection on every other day after P. acnes induction for 3 days	3 days	Suppressed NLRP3 inflammasome formation by blocking deubiquitylation of NLRP3	Yan et al. (2021)

APAP, acetaminophen; CCl4, carbon tetrachloride; AMPK, Adenosine 5′-monophosphate (AMP)-activated protein kinase; SIRT3, sirtuin3; PPARα, Peroxisome Proliferator Activated Receptor α; DCA, deoxycholic acid; EGR1, Early Growth Response 1; ANIT,α-naphthyl isothio-cyanate; TAA, thioacetamide; SIRT1, sirtuin1; FXR, Farnesoid X receptor; EE, 17 -ethinyl estradiol; ICP, intrahepatic cholestasis of pregnancy; MMP-2, Matrix metalloproteinase-2; MMP-9, Matrixmetalloproteinase-9; CLP, cecal ligation and puncture; NF-κB, nuclear factor kappa-B; TLR4, Toll-like receptor 4; 5-LOX, 5-Lipoxygenase; IL-6, Interleukin-6; LPS, lipopolysaccharides; P. acnes/LPS, Propionibacterium acnes/lipopolysaccharides; NLRP3, NOD-like receptor protein 3.

As the number and variety of drug applications grow rapidly, drug-induced liver injury (DILI), especially acetaminophen (APAP) overdose, is becoming the most common cause of acute liver failure (ALF) in America and Europe (Shehu et al., 2017). A series of studies demonstrated that pretreatment with celastrol attenuated the APAP-induced oxidative stress and inflammation by raising antioxidant enzyme activities such as glutathione peroxidase (GPx), glutathione reductase (GR), catalase, and superoxide dismutase (SOD) activities both *in vitro* and *in vivo* (Abdelaziz et al., 2017; Jannuzzi et al., 2018). CCl4 as another hepatotoxic chemical could induce liver injury by inflammatory activation and antioxidant enzymes inhibition (Zhang et al., 2017b). Celastrol might also suppress inflammation in CCl4 induced liver injury rat by activating AMPK-SIRT3 signaling (Wang et al., 2020). In the latest research, Li and his colleagues found that celastrol could eliminate oxidative stress and reduce the release of proinflammatory cytokines by activating PPARα signaling pathway in CCl4 intoxicant mice (Zhao et al., 2020). However, celastrol itself as a drug also has toxicity, the balance between the liver protection and liver injury needs to be further studied, and new structure modification and formulation should be developed to reduce toxicity and enhance efficacy.

Cholestasis is a pathophysiological process caused by bile secretion and excretion disorders. It is manifested as excessive accumulation of bile components such as bile acid, cholesterol, and bilirubin in the liver and systemic circulation, causing damage to liver cells and whole body. Long-term continuous cholestasis will progress to liver fibrosis and even cirrhosis (Zollner and Trauner, 2008; Padda et al., 2011). Li and his colleagues demonstrated that celastrol ameliorated cholestatic liver injury induced by α-naphthyl isothiocyanate (ANIT) and thioacetamide (TAA) through activation of SIRT1-FXR signaling pathway, while the hepatoprotective effects of celastrol were abrogated in FXR deficiency or SIRT1 inhibition mice (Zhao et al., 2019b). In addition to that, 5 mg/kg/day celastrol remarkably alleviated intrahepatic cholestasis of pregnancy via downregulating matrix metalloproteinase (MMP-2 and MMP-9) and total bile acid in pregnant rats (Guo et al., 2019).

Sepsis is a serious clinical syndrome caused by many organisms including bacteria, viruses, and fungi, which might lead to liver injury (Strnad et al., 2017). Cecal ligation and puncture (CLP) model and lipopolysaccharide (LPS) inducement is often used to mimics sepsis (Zhang et al., 2014; Xiong et al., 2017; Li et al., 2020). Celastrol attenuated CLP induced hepatic dysfunction in rats by inhibiting toll-like receptor-4 (TLR-4)/NF-κB (El-Tanbouly et al., 2017). In addition, celastrol pretreatment was found to attenuate propionibacterium acnes/lipopolysaccharides induced liver damage via NLRP3 inflammasome suppression and inflammatory inhibition (Yan et al., 2021). On the contrary, celastrol aggravated liver injury by exacerbation of inflammatory and elevation of oxidative stress in LPS intoxicant mice (Wu et al., 2018). The contradictory results were attributed to the different dosage of LPS and celastrol, and it is hard for high dosage of celastrol to reverse the severe liver failure that resulted from high dose of LPS, and the toxicity of celastrol should be taken into consideration in treating sepsis.

In short, these data suggested that celastrol is a potential candidate for treatment of hepatic injury, mainly owing to its ability to inhibit inflammation and oxidative stress and recover bile acid homeostasis. While protecting the liver injury, the toxicity of celastrol should be also taken into consideration to avoid side effects.

### Celastrol and Liver Cancer

Hepatocellular carcinoma (HCC) as one highly malignant cancer remains a global menace. Effective systemic treatment of HCC is impeded by its complicated molecular pathogenesis, high rate of metastasis, recurrence, and chemo-resistance. It is imperative to explore effective therapy strategies for liver cancer. Natural medicine with long history of clinical use exhibited great potential in anti-cancer due to its high efficiency and availability, as well as low side effects (Hu et al., 2013). Numerous studies have shown that celastrol exerts anticancer effects by inhibiting their proliferation, metastasis, and inflammatory properties depending on modulating a variety of signaling pathways (Kashyap et al., 2018).

#### Anti-Proliferation and Pro-Apoptotic Effects *In Vitro*

Celastrol arrested cell cycle and promoted apoptosis through suppression of STAT3/JAK2 signaling, PI3 K/Akt signaling, and ER-stress/UPR signaling in different hepatocellular cell lines (Table 4) (Chen et al., 2011; Rajendran et al., 2012; Li et al., 2013; Wei et al., 2014; Li et al., 2015; Ma et al., 2017; Ren et al., 2017; Du et al., 2020; Kun-Ming et al., 2020). In addition, celastrol inhibited hepatoma cell line growth and proliferation via arresting cell cycle in sub-G1 (Rajendran et al., 2012) and G2/M phase (Chen et al., 2011; Ren et al., 2017). Further studies showed that celastrol reduced the expression of c-myc and cyclin D1 which was closely related to cell cycle arrest of tumor cells *in vivo* on a time- and dose-dependent manner (Rajendran et al., 2012; Ma et al., 2017). Mechanistic studies revealed that celastrol could impede liver cancer through downregulation of HSP90 proteins, activation of c-Jun NH2 terminal kinase (JNK), and weakened MRC complex I activity (Chen et al., 2011). Apart from that, celastrol could also inhibit the migration, invasion, and metastasis of liver cancer by inhibiting the ROCK2-mediated phosphorylation of ezrin at Thr567 (Du et al., 2020), suppressing NF-κB and Akt activity, and downregulating miR-224, MMP-2, and MMP-9 (Li et al., 2013).

**TABLE 4:** Anti-cancer effects of celastrol *in vitro*.

Cell line	Effect of celastrol	Mechanism	Doses	Time	Reference
HepG2, Bel-7402	Anti-proliferation, induce apoptosis	Downregulated the expression of E2F1	2.5 and 5 µM	24 h, 48 h	Ma et al. (2017)
HepG2, Bel-7402	Anti-proliferation, induce apoptosis	Induced autophagy and ER stress, lead to G2/M phase arrested	1.25, 2.5, and 5 µM	24 h	Ren et al. (2017)
Bel-7402	Anti-proliferation, induce apoptosis	Promoted cytochrome c release, increase the expression of cleaved caspase-9, caspase 3 and the ratio of Bax/Bcl-2	0.78, 1.56, and 3.12 μg/ml	24, 48, and 72 h	Li et al. (2015)
HepG2	Anti-proliferation, anti-migration	Inhibited the CXCR4 mediated PI3K/Akt pathway, lead to sub-G0 phase arrested	0.1, 0.3, 0.625, and 1 µM	24 h	Kun-Ming et al. (2020)
HepG2	Anti-proliferation, induce apoptosis	Induced ROS accumulation and G2-M phase blockage	2, 4, and 6 µM	24 h	Chen et al. (2011)
HepG2	Anti-metastasis	Repressed NF-κB and Akt activity, downregulate the expression of miR-224, MMP2, and MMP9	0.1, 0.5, 1 µM	18 h	Li et al. (2013)
Huh7, Hep3B	Anti-proliferation, Anti-migration, Anti-invasion and Enhanced Apoptosis	Repressed circ_SLIT3 and Bcl-2, raised the Bax expression, impeded circ_SLIT3/miR-223-3p/CXCR4 signaling	0.5, 1 µM	48 h	(Si et al., 2013; Kun-Ming et al., 2020)
C3A	Anti-proliferation, induce apoptosis, anti-metastasis	Modulated STAT3 activation with the inhibition of c-Src, JAK1, and JAK2 activation; downregulate the expression of cyclin D1, Bcl-2, Bcl-xL, survivin, Mcl-1, and VEG, caused sub-G1 phase arrested	2.5, 5, and 10 µM	24, 48, and 72 h	Rajendran et al. (2012)
MHCC97H	Anti-migration	Inhibited the ROCK2 mediated phosphorylation of ezrin at Thr567	0.5 µM	4 and 8 h	Du et al. (2020)

E2F1, E2F transcription factor 1; ER, endoplasmic reticulum; Bcl-2, B-cell lymphoma-2; Bax, BCL2- Associated X,; CXCR4, C-X-C motif chemokine receptor 4; PI3K, phosphatidylinositol-3 -kinase; AKT, protein kinase B; ROS, reactive oxygen species; NF-κB, nuclear factor kappa B; MMP2, Matrix metallopeptidase 2; MMP9, Matrix metallopeptidase 9; ccirc_SLIT3, circRNA, slit guidance ligand 3; JAK1, Janus kinase1; JAK2, Janus kinase2; Mcl-1, Myeloid Cell Leukemia Sequence 1; VEGF, vascular endothelial growth factor; ROCK2, Rho Associated Coiled-Coil Containing Protein kinase 2.

#### Tumor Suppression Effects *In Vivo*

As show in Table 5 (Chen et al., 2011; Rajendran et al., 2012; Wei et al., 2014; Chang et al., 2016; Kun-Ming et al., 2020; Saber et al., 2020; Si et al., 2021), studies have manifested that celastrol exhibits anticancer activity against a variety of liver cancer animal models such as HCC patient-derived xenografts BALB/cJ mice (Wei et al., 2014), different hepatocellular carcinoma cells derived xenografts mouse models (Rajendran et al., 2012; Ma et al., 2014; Ren et al., 2017; Si et al., 2021), and DEN induced HCC rats/mice (Chang et al., 2016; Saber et al., 2020) with dosage of 1–10 mg/kg for 3–10 weeks. Celastrol (4 mg/kg) prevented tumor proliferation and increased apoptosis via inhibition of STAT3/JAK2 signaling pathway in PLC/PRF5 cells derived xenografts HCC mice (Rajendran et al., 2012). Recently, different studies validated that inhibition of circ_SLIT3/miR-223-3p/CXCR4 signaling is involved in the anti-HCC activity of celastrol both *in vitro* and *in vivo* (Kun-Ming et al., 2020; Si et al., 2021). Meanwhile, celastrol has been demonstrated to decrease the hepatic lesions and elevation of serum alanine aminotransferase (ALT), glutamic oxalacetic transaminase (AST), alkaline phosphatase (ALP), and alpha fetoprotein (AFP) in diethylnitrosamine (DEN)-induced hepatocellular carcinoma (HCC) rats, due to activation of mitochondrial apoptosis induced by p53 (Chang et al., 2016; Saber et al., 2020). Consistent with this, combination therapy with celastrol (2 mg/kg) and metformin (200 mg/kg) not only attenuated hepatic injury with elevated liver enzymes but also decreased NLRP3 mediated NF-κB signaling to suppress anti-apoptotic processes in mice with DEN-induced HCC (Saber et al., 2020). Meanwhile, Jiang and his colleagues found that synergistic use of celastrol and PHA665752 (a c-Met inhibitor) significantly inhibited cell growth, migration in c-met deficient Huh7 cells, and Huh7 xenografts nude mice (Jiang et al., 2013). Besides that, celastrol could enhance the activity of anti-liver cancer drugs including sorafenib, lapatinib, and ABT-737 by cell growth inhibition and apoptosis induction (Zhu et al., 2012; Yan et al., 2014; Zhang et al., 2019). These results indicated that celastrol not only has good efficiency on killing cancer cells but also can improve the anti-cancer ability of first-line drugs for tumor therapy.

**TABLE 5:** The anti-tumor effects of celastrol *in vivo*.

Cancer model (animal)	Dose and formulation	Treatment period	Tumor volume	Mechanism	Reference
HCC patient-derived xenografts (BALB/cJ mice)	4 mg/kg/day by intravenous injection	3 weeks	Reduce 2–5 fold	Pro-apoptosis, anti-proliferation through inhibited phosphorylation of protein kinases in the Raf/MEK/ERK and PI3K/AKT/mTOR signaling pathways	Wei et al. (2014)
H22 cells derived xenografts (female BALB/c mice)	1 and 2 mg/kg/day by i.p injection	3 weeks	Reduce 2–4 fold	Induced of ER stress and apoptosis	Ren et al. (2017)
Hep3B cells derived xenografts (athymic nu/nu female mice)	3 and 10 mg/kg/day by oral administration three times a week	5 weeks	Reduce 2–2.5 fold	Reduced the hypoxia-induced accumulation of HIF-1α protein, inhibited angiogenesis, invasion, and metastasis	Ma et al. (2014)
PLC/PRF5 cells derived xenografts (athymic nu/nu female mice)	1 and 2 mg/kg/day by i.p injection	3 weeks	Reduce 1.5–2.5 fold	Antiproliferative and proapoptotic effects through suppression of STAT3 signaling	Rajendran et al. (2012)
Hep3B cells derived xenografts (athymic nu/nu female mice)	2 mg/kg/day by i.p injection every 5 days	30 days	Reduce 2–2.5 fold	Inhibited circ_SLIT3/miR-223-3p/CXCR4 signaling	Si et al. (2021)
DEN induced HCC in rats	2, 4, and 8 mg/kg/day by oral administration	10 weeks	Reduce 1.5–3 fold	Activated mitochondrial apoptosis pathway	(Chang et al., 2016; Saber et al., 2020)

i.p, intraperitoneal; Raf, rapidly accelerated fibrosarcoma; ERK, Extracellular-signal-regulated kinases; MEK, Mitogen-activated protein kinase/ERK kinase; PI3K, phosphatidylinositol-3-kinase; AKT, protein kinase B; mTOR, mammalian target of rapamycin; STAT3, signal transducer and activator of transcription 3; HIF-1α, hypoxia-inducible factor, circ_SLIT3, circRNA slit guidance ligand 3; CXCR4, C-X-C motif chemokine receptor.

#### The Role of Hypoxia-Inducible Factor in Anti-Tumor Activity of Celastrol

Hypoxia was one of most important regulators in liver cancer progression. Hypoxia-inducible factor (HIF-1), a nuclear transcription factor, might be activated under hypoxia and specifically regulated oxygen and metabolic homeostasis (Balamurugan, 2016). HIF-1/2α protein is highly expressed in human HCC tissues, and correlates with tumor invasion and metastasis in HCC patients (Bangoura et al., 2004; Wong et al., 2014). In addition to antiproliferative and proapoptotic effects, celastrol repressed tumor growth, angiogenesis, invasion, and metastasis through reduced hypoxia-induced accumulation of HIF-1α protein (Ma et al., 2014). Ma et al. demonstrated that celastrol inhibited the hypoxia-induced accumulation of HIF-1α protein due to the downregulation of mTOR/p70S6K/EIF4E and ERK1/2 phosphorylation in Hep3B cells derived xenografts nude female mice, and the expression of angiogenesis related factors-vascular endothelial growth factor (VEGF) and erythropoietin (EPO) are both prevented (Ma et al., 2014). However, this result was challenged by Han and his co-workers, who found that celastrol promoted HIF-1α protein accumulation by inducing ROS and activating Akt/p70S6K signaling in HepG2 cells under hypoxia, and celastrol suppressed cancer cell growth by promoting mitochondrial autophagy and apoptosis *in vitro* (Han et al., 2014). The difference in hypoxia exposure time (16 h in Han et al.’s work vs. 6 h in Ma et al.’s study) of HepG2 cells might contribute to the discrepant results on HIF-1a expression for celastrol in the above studies.

**FIGURE 3:**
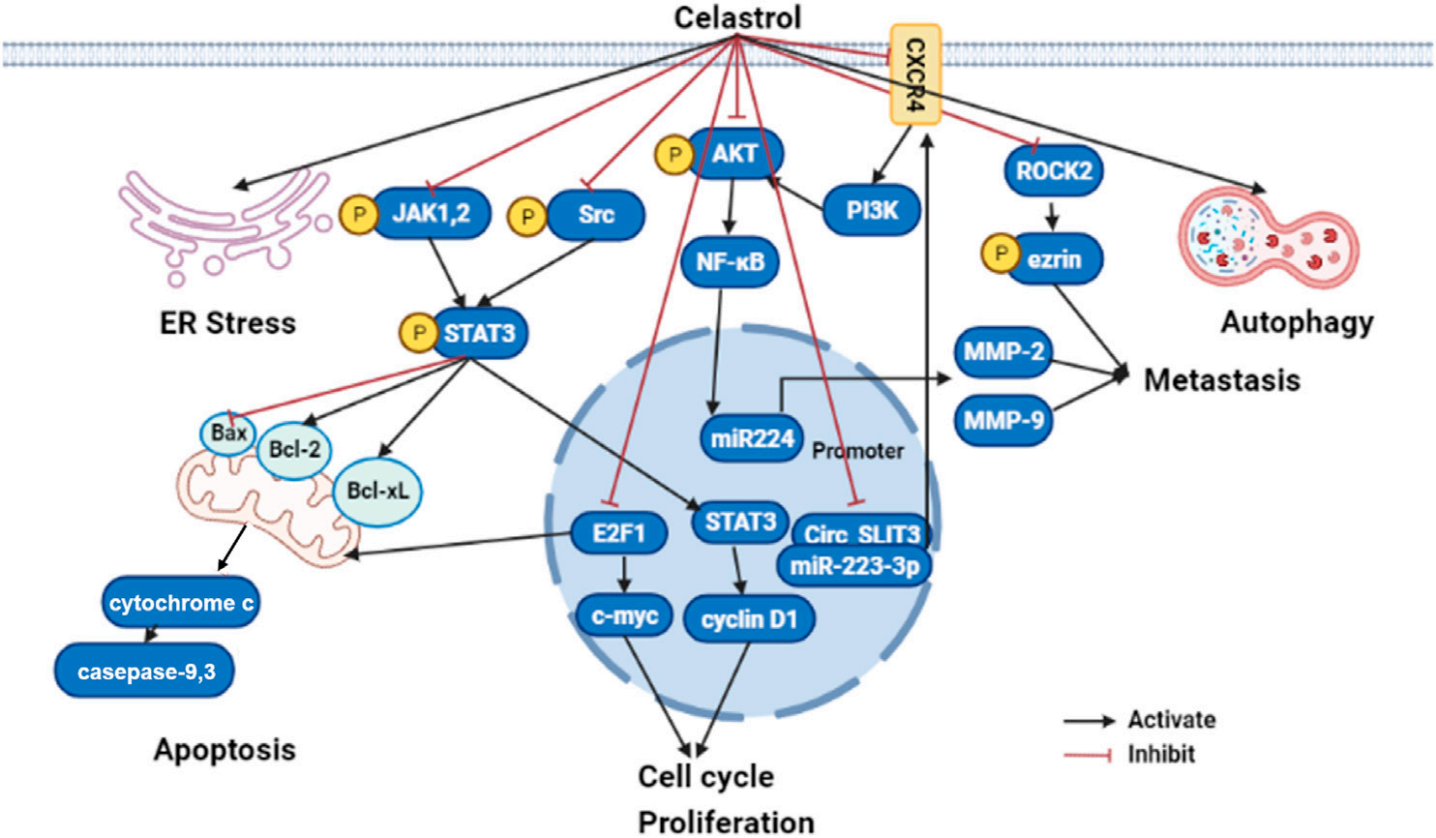
The molecular mechanisms of celastrol in liver cancer (edited by Biorender software). Celastrol exert anticancer effect by restraining their cell growth, metastasis, and inflammatory response, while boosting ER stress, apoptosis, and autophagy depending on modulated different signaling pathways. Firstly, celastrol can activate cancer cell apoptosis through inhibiting JAK2 and STAT3 phosphorylation, then downregulated BCL-2 family proteins (Bcl-2 and Bcl-xl) and upregulated caspase family proteins (caspase 3 and caspase 9). Secondly, celastrol repressed tumor cell proliferation by suppressing cyclin D1 and c-myc through E2F1 and STAT3; Lastly, celastrol prohibited tumor metastasis by NF-κB signaling pathway modulated MMP-2 and MMP-9 through the CXCR4-related signaling pathway, as well as the ROCK2-mediated phosphorylation of ezrin at Thr567. ER, endoplasmic reticulum; JAK1, Janus kinase1; JAK2, Janus kinase2; STAT3, signal transducer and activator of transcription 3; Bcl-2, B-cell lymphoma-2; PI3K, phosphatidylinositol-3-kinase; AKT, protein kinase B; NF-kB, nuclear factor kappa B; MMP2, Matrix metallopeptidase 2; MMP9, Matrix metallopeptidase 9; circ_SLIT3, circRNA slit guidance ligand 3; ROCK2, Rho Associated Coiled-Coil Containing Protein kinase 2.

Summarily, celastrol might be a potential therapeutic candidate for the treatment of liver cancer due to reduction of the oxidative stress, inhibition of inflammatory response, and activation of mitochondrial autophagy. Although the studies were performed both *in vitro* and *in vivo*, the clinical research still should be performed to evaluate whether celastrol could apply as effective drug or adjuvant drug to further improve clinical outcomes. The mechanisms of celastrol on anti-cancer are summarized in Figure 3.

## Limitations and Strategies for the Use of Celastrol

Although celastrol exhibited many benefits on various liver diseases, the shortcomings of celastrol limited its clinical transformation. On the one hand, the poor water solubility of celastrol (13.25 ± 0.83 μg/ml at 37°C) results in low oral bioavailability (17.06% in rat) (Qi et al., 2014), and more researches should be conducted on improving its water solubility and oral bioavailability. On the other hand, the side effects of celastrol and narrow therapeutic window of dose also could be a big problem for its clinical use. While 0.25 mg or 0.5 mg/kg/d, celastrol (i.p.) attenuated the LPS induced inflammation (Yan et al., 2021), 1.5 mg/kg/d, celastrol (i.p.) (Wu et al., 2018) aggravated the liver injury induced by celastrol. Beyond that, it was insufficient for CYP450s to metabolize celastrol, which led to the hepatotoxicity. In addition to damaging the liver, celastrol also has side effects on kidney (Wu et al., 2018), cardiovascular system, and hematopoietic system (Zhang et al., 2012). New modification and formulation are urgently needed to overcome the shortcomings and improve its efficacy.

Hopefully, new approaches have been reported to improve the water solubility and bioavailability of celastrol. Lipid-based nanocarriers (including the self-microemulsifying drug delivery system [SMEDDS] [Chen et al., 2012], nanostructured lipid carrier [NLC], and phytosomes) (Freag et al., 2018), polymer-based nanocarriers like PEG-PCL (poly(ethylene glycol)-poly(ɛ-caprolactone) copolymers) nanoparticles (Zhao et al., 2019a), and galactosylated liposomes (Chen et al., 2020b) markedly enhanced the water solubility and oral bioavailability of celastrol. Combination with other drugs can be an effective approach to reduce the toxicity and enhance the efficacy of celastrol. Currently, celastrol combined with chemotherapeutic agents, tumor necrosis factor superfamily, active ingredients of TCM, ionizing radiation, nucleic acid, and other therapies have been applied to the treatment of various cancers *in vitro* (Shi et al., 2020). The combined therapy reduced the dosage of celastrol and the related adverse effect, while the efficacy of celastrol improved. In addition, structural modifications will be a promising way to reduce the toxicity and improve the water bioavailability of celastrol, and the correlation between structure and toxicity have been well studied (Hou et al., 2020). Chemical modifications of celastrol mainly focused on the A/B ring and C-20 carboxyl group. In addition to chemical modifications, Chang et al. demonstrated that the glycosylation of celastrol formed by biotransformation showed over 53-fold higher water solubility and exhibited 50-fold less toxicity than celastrol *in vivo* (Chang et al., 2021), which indicated that biotransformation or biosynthesis might be a useful strategy for the clinical use of celastrol.

## Conclusion and Perspectives

A large number of studies have shown that celastrol might be a potential drug for different liver diseases treatment. In this review, we summarized the recent studies about the protective effects and mechanisms of celastrol in several liver diseases, including MAFLD, and it caused liver injury, or liver cancer. The liver protection effect could be attributed to modulating metabolic balance, inhibiting inflammatory response, and activating autophagy by altering different cellular pathways (Tables 1–5). Although many researchers elucidated the molecular mechanisms of celastrol in liver diseases, the direct targets of celastrol are unknown. The SPR assay, affinity chromatographic methods, and other methods might be used to explore the direct interaction between celastrol and the potential targets. At the same time, with the development of omics, celastrol regulates gut microbes and liver metabolism to exert hepatoprotective effects required further investigated.

Moreover, although accumulating evidence demonstrated that celastrol grants tremendous potential in liver diseases at cellular or animal levels, the clinical studies of celastrol in liver diseases are very rare. Comprehensive studies on efficacy, safety, and toxicity in humans urgently need to be carried out. Besides, the poor water solubility, low bioavailability, and narrow therapeutic window of dose also limited the clinical application of celastrol. New modifications and formulations are needed to overcome the shortcomings and improve its efficacy. It is foreseeable that celastrol and its derivatives will be a promising drug for the treatment of liver diseases.
